# Prophylactic Effects of Levamisole and Vitamin E on Phenobarbital-induced Cleft Palate and Spina Bifida in Rat Embryos

**Published:** 2011

**Authors:** Mahmood Khaksary Mahabady, Hossein Najafzadeh Varzi

**Affiliations:** a *Department of Anatomy and Embryology, Faculty of Veterinary Medicine, Shahid Chamran University, Ahwaz, Iran.*; b * Department of Pharmacology, Faculty of Veterinary Medicine, Shahid Chamran University, Ahwaz, Iran.*

**Keywords:** Phenobarbital, Levamisole, Cleft palate, Spina bifida, Teratogenicity, Rat

## Abstract

There are many reports that show the teratogenic effects of phenobarbital can be decreased by stimulation of maternal immune system. Therefore, in this study, the prophylactic effects of levamisole and vitamin E on teratogenic effects of phenobarbital were compared. This study was performed on 20 pregnant rats that were divided into four groups. Control group received normal saline and test groups received phenobarbital (120 mg/kg), phenobarbital (120 mg/kg) plus levamisole (10 mg/kg) and phenobarbital (120 mg/kg) plus vitamin E (100 mg/kg) intraperitoneally at 9-11th days of gestation, respectively. Fetuses were collected at 20th day of gestation and after determination of weight and length; they were stained by Alizarin red - Alcian blue method. Cleft palate and spina bifida incidence were 66.66% and 69.44% in fetuses of rats that had received only phenobarbital. Cleft palate and spina bifida incidence were 65.45% and 38.18% had in the group which had received phenobarbital plus levamisole. However, Cleft palate and spina bifida incidence were 54.54% and 27.27% in the group which had received phenobarbital plus vitamin E. The arithmetic means of the weight and length of fetuses the rats that had received levamisole and vitamin E were significantly greater than that of those that had received only phenobarbital. Vitamin E had a greater prophylactic effect than levamisole on the incidence of phenobarbital-induced cleft palate and spina bifida. However, the difference was not significant.

## Introduction

Phenobarbital is one of the major drugs in the treatment of epilepsy. It is also a hypnotic and sedative drug (1-3). Several studies show that the stimulation of maternal immune system can decrease or prevent drug- induced embryonic abnormalities (4-6). For example, macrophage activation decreases incidence of cleft palate and digital and tail anomalies in fetuses of mice that received urethane and methylnitrous urea (5). Interferon gamma reduces urethane - induced cleft palate and granulocyte-colony stimulating factor decreases cyclophosphamide -induced distal limb abnormalities in mice (7).

Levamisole as antinematodal has immunomodulatory effect in human and animals. This drug stimulates T and B cells proliferation and antibodies production (8). Khaksary Mahabady *et al*. (2006) observed beneficial effect of levamisole on decreasing phenytoin-induced cleft palate in mice (9).

Vitamin E, a natural antioxidant, is believed to prevent diseases associated with oxidative stress (10). Vitamin E is considered safe in pregnancy, although experiments evaluating the safety of high-doses of vitamin E in pregnancy have not been reported (11).

In the present study, the preventive effect of vitamin E and levamisole on phenobarbital-induced cleft palate and spina bifida in rats was compared.

## Experimental

Phenobarbital (Sigma, USA), levamisole (Rouz daru, Iran) and vitamin E (Darupakhsh, Iran) were purchased from commercial sources.

Male and female healthy rat of Wistar strain, 3-4 month old, weighing 200-250 g were purchased (Razi Institute, Karadje, Iran) and housed individually (males) or at 10 per polycarbonate cage (female) for a 2-week acclimatization period. The rats were fed *ad libitum *standard laboratory pellet (Pars khurakdam, Shushtar, Iran.) and tap water. A 12 h light: 12 h dark cycle was maintained. Room temperature was maintained at 23 ± 2°C with a relative humidity of 45-55%.

Female rats were mated overnight with males. The vaginal plug was assumed as first day of gestation (GD1). Pregnant animals were divided into four groups (n = 5) and treated as follows:

The control group received normal saline (5 mL/kg), the test groups received phenobarbital (120 mg/kg) (12), phenobarbital (120 mg/kg) plus levamisole (10 mg/kg) (9), and phenobarbital (120 mg/kg) plus vitamin E (100 mg/kg) (13) intraperitoneally, respectively.

The animals were sacrificed by cervical dislocation on the 20th day of gestation and their fetuses were collected and numbered, then their weight and length (crown- rump length) were measured. The fetuses were stained by Alizarin red-Alcian blue method (14) and examined by stereomicroscope for cleft palate and spina bifida. The incidence of cleft palate and spina bifida were determined.

Statistical significance was determined using SPSS program and comparisons were made by one way analysis of variance (ANOVA) and chi-square test. The minimum level of significance was p < 0.05.

## Results and Discussion

**Figure 1:**
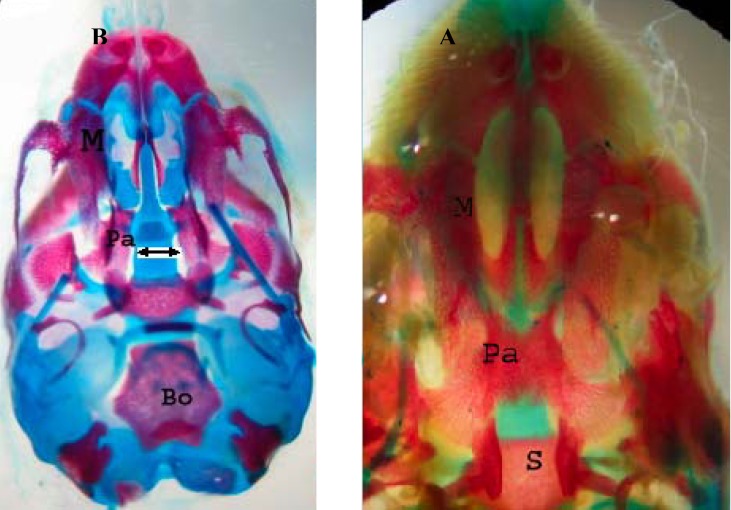
Ventral view of skull of gestation 20th day fetal rat (A). Normal palatine bone (B). Cleft palate induced by phenobarbital (arrow) which stained with Alizarin red- Alcian blue. M: Maxilla, Pa: Palatine, S: Sphenoid, Bo: Basioccipital

**Figure 2:**
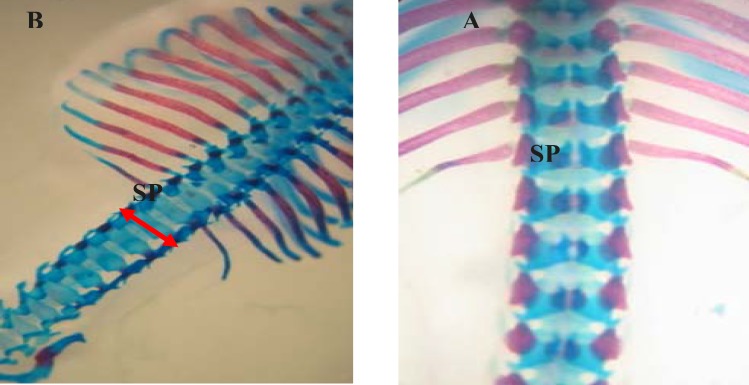
Dorsal view of vertebral column of gestation 20th day fetal rat. (A) Normal (B) Spina bifida (arrow) induced by phenobarbital which stained with Alizarin red- Alcian blue. SP: Spinous process

In the control group, palatal closures of fetuses were normal (Figure 1A). Phenobarbital induced cleft palate and spina bifida at 66.66%, 69.44% incidence, respectively (Figure 1B, 2B).

**Figure 3:**
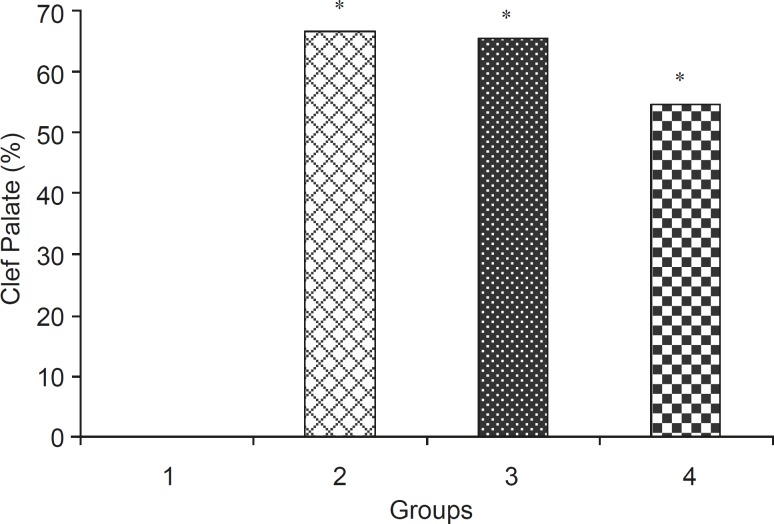
Incidence (% + SEM) of cleft palate in normal saline and test groups, 1: Normal saline (5 mL/kg), 2: Phenobarbital (120 mg/kg IP), 3: Phenobarbital + levamisole (10 mg/kg IP), 4: Phenobarbital + vitamin E (100 mg/kg IP), n = 5, * Significant difference with normal saline group (p < 0.05)

**Figure 4:**
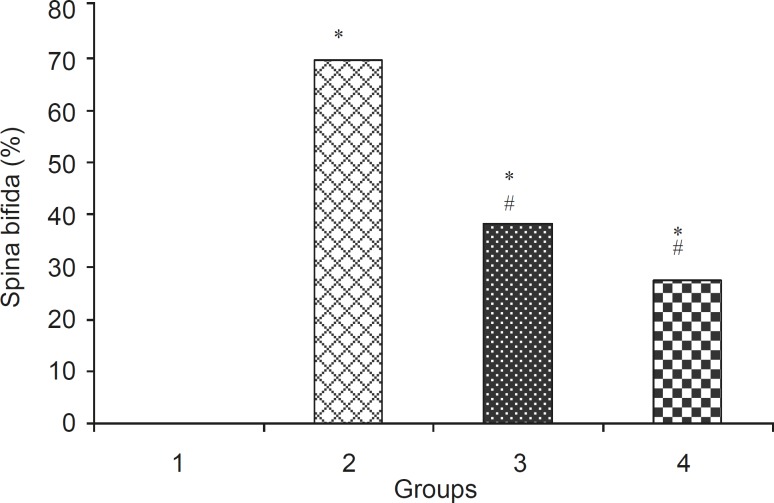
Incidence (% + SEM) of spina bifida in normal saline and test group. 1:Normal saline (5 mL/kg), 2: Phenobarbital (120 mg/kg IP), 3: Phenobarbital+levamisole 4: Phenobarbital + vitamin E (100 mg/kg IP), n = 5, *Significant difference with normal saline group, # Significant difference with phenobarbital group (p< 0.05).

**Figure 5:**
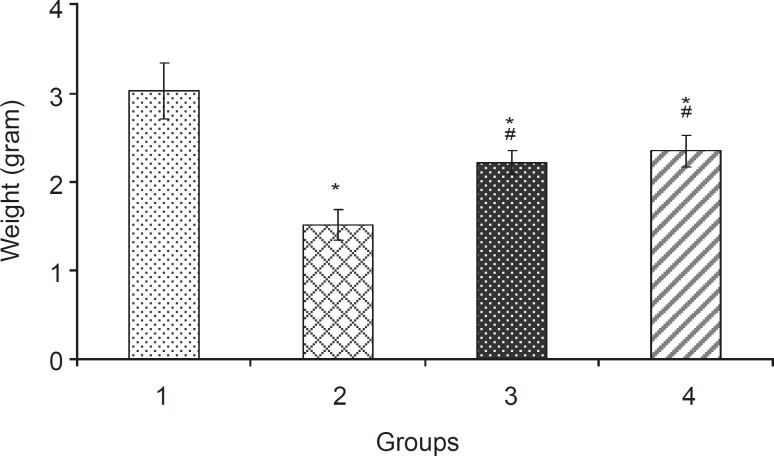
Weight (mean ± SEM) of fetuses in different groups: 1: Normal saline (5 mL/kg), 2: Phenobarbital (120 mg/kg IP), 3: Phenobarbital + levamisole (10 mg/kg IP), 4: Phenobarbital + vitamin E (100 mg/kg IP), n = 5, * Significant difference with normal saline group, # Significant difference with Phenobarbital group (p<0.05).

**Figure 6:**
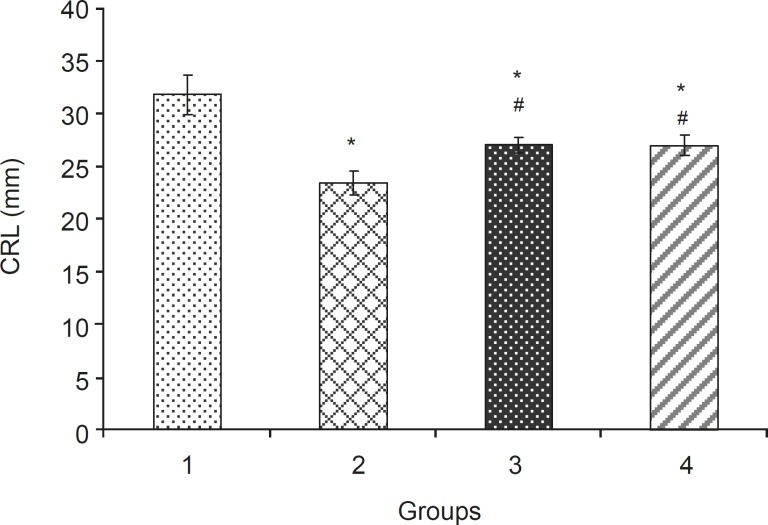
Crown rump length (mean ± SEM) of fetuses in different groups: 1: Normal Saline (5 mL/kg), 2: Phenobarbital (120 mg/kg IP), 3: Phenobarbital+levamisole (10 mg/kg IP), 4: Phenobarbital + vitamin E (100 mg/kg IP), n = 5, * Significant difference with normal saline group (p < 0.05), # Significant difference with phenobarbital group (p < 0.05).

Levamisole reduced incidence of phenobarbitalinduced cleft palate and spina bifida to 65.43%, 38.18%, respectively (Figure 3, 4). Mean weight and length (CRL) were significantly (p < 0.001) decreased in the group that had received only phenobarbital. The weight and length arithmetic means of the groups that had received levamisole and vitamin E were greater than those of the group that had received only phenobarbital (Figure 5, 6). No intrauterine death of the animals treated was observed.

Several studies have reported that the maternal immune system stimulation can reduce teratogenic anomalies. The mechanism of this observation has remained unclear. However, this reduction is believed to be due to modulation of the fetal gene expression (4).

 In the present study, both vitamin E and levamisole reduced the incidence of cleft palate formation. Vitamin E decreased incidence of cleft palate formation more than levamisole, but the difference was not significant. Enhancing antioxidative effects can protect fetuses against phenytoin teratogenicity (15).

Sharova L. *et al. *showed that interferon-gamma and Freund’s complete adjuvant reduced

severity of the urethane-induced cleft palate in mice (16). Torkinsky *et al. *(1997) reported that

maternal immune system stimulation in diabetic mice, which showed a high spontaneous rate of

cleft palate, decreased in malformed fetuses, significantly (17).

 Sullivan *et al. *(1977) evaluated the teratogenic activity of phenobarbital in mice. They observed that phenobarbital can produce teratogenicity in fetuses of mice (12). They observed fetal defects similar to those we observed in our study, including cleft palate. These anomalies were decreased by levamisole (10 mg/kg).

Levamisole is an anthelmintic agent that also apparently enhances immune responsiveness. It is believed that levamisole mediates immune function of T- cells and stimulates phagocytosis by monocytes. Its immunostimulating effects are greater in immune - compromised animals (8). In addition, levamisole showed antitumor effect in mice (18). In the present study, the effect of levamisole is probably related to immunologic response. 

Administration of vitamin E to pregnant diabetic animals decreases the rate of embryonic malformations, increases their body weight and accelerates their maturation (13). Boskvic *et* *al*. reported that consumption of high doses of vitamin E during the first trimester of pregnancy was not associated with an increased risk for major malformations, but may be associated with a decrease in birth weight (19). On the other hand, supplementing the diet of ewes resulted in a significant increase in lamb birth weight (20).

In conclusion, phenobarbital probably influences the immune system, producing teratogenic effects including cleft palate and spina bifida. The effects of Phenobarbital on immunosuppression are mediated indirectly by inducing oxidative stress (2, 3).

 On the other hand, vitamin E is more effective than levamisole in decreasing incidence of phenobarbital -induced cleft palate and spina bifida in fetuses of rat, but the effect was not significant.
